# Malignant melanoma associated with a blue naevus: a case report

**DOI:** 10.1186/1757-1626-1-433

**Published:** 2008-12-31

**Authors:** Pasquale Mellone, Alexander Bianchi, Emanuele Dragonetti, Raffaele Murace, Paolo Persichetti, Alfonso Baldi

**Affiliations:** 1 Department of Biochemistry, Sect. Pathology, Second University of Naples, Naples, Italy; 2 Rome American Hospital, Rome, Italy; 3 Paideia Hospital, Rome, Italy; 4 Campus BioMedico University, Rome, Italy

## Abstract

**Introduction:**

Combined malignant naevi are characterised pathologically by the association of a melanoma with one or more different types of benign melanocytic naevi in a single lesion.

**Case Presentation:**

We show here a case of malignant combined naevus made up of a blue naevus and a melanoma, presenting as a slowly progressing and asymptomatic pigmented lesion on the trunk of a 35-year-old man. Dermoscopic examination was not conclusive for a malignant lesion, showing only an atypical brown pigment network. The definitive diagnosis was reached only at the hystopathological examination.

**Conclusion:**

This finding suggests that combined naevi should be always excised and histologically examined to achieve a correct diagnosis and avoid risk of misclassification.

## Introduction

Combined naevi are unusual pigmented skin lesions that represent less than 1% of all biopsed melanocytic naevi [1]. They are characterised by the presence of two or more different types of melanocytic naevi in a single lesion, being the association of a blue nevus with a Clark or Spitz nevus the most common entity. Dermoscopic analysis of combined naevi commonly shows a multicomponent pattern with the typical findings of each lesion [2]. However, in several cases, the differential diagnosis between a benign combined naevus and melanoma can be very difficult at the dermoscopic evaluation [3].

## Case presentation

**Figure 1:**
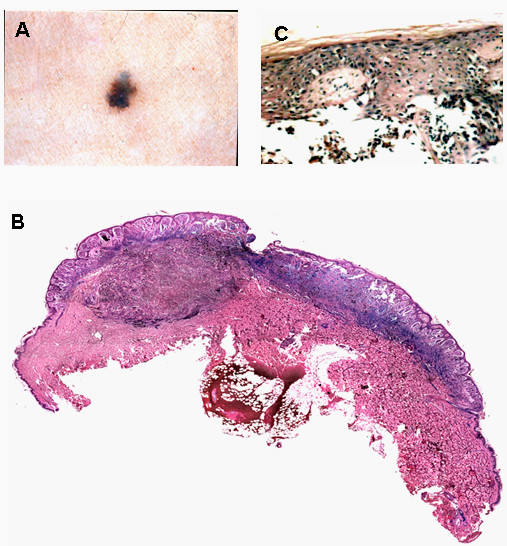
**A) Dermoscopic appearance of the malignant combined nevus showing a multicomponent pattern made up of an homogeneous central steel or blu-gray pigmentation with an irregular border surrounded by an atypical brown pigment network**. B) Microscopical appearance of the pigmented lesion showing features of a combined malignant naevus (blue naevus + melanoma), composed of a malignant melanoma, superficial Clark's level III invasion, arising in association with a blue naevus in the deep dermis (Haematoxylin and Eosin, original magnification × 5). C) A detail at greater magnification of figure 1B, showing the invasion of the epidermis by the neoplastic melanocytes (Haematoxylin and Eosin, original magnification × 20).

A 35-year-old man presented with a 1-year slowly progressing and asymptomatic pigmented lesion of the trunk. Physical examination revealed an irregular, oval, blue-brown plaque with the dimensions of 5.5 × 3.3 mm. Dermoscopic examination revealed a multicomponent pattern made up of an homogeneous central steel or blu-gray pigmentation with an irregular border surrounded by an atypical brown pigment network (Figure 1A). This pattern was suggestive of a combined naevus, even if it was not possible to exclude the diagnosis of melanoma due to the obscuration of the pigment network by the blue component of the lesion. The excised biopsy specimen of the tumor was fixed in 10% buffered-formalin and paraffin embedded. Sections of 5 μ were stained with haematoxylin-eosin, and haematoxylin-van Gieson. Other sections were stained with immunohistochemical procedure, using avidin-biotin peroxidase complex (ABC; Dako, Carpinteria CA) to the following antibodies from Dako: anti-HMB45 and anti-Mart1. Light microscopic examination showed features of a combined malignant naevus (blue naevus + melanoma), composed of a malignant melanoma, superficial Clark's level III invasion, arising in association with a blue naevus made up of round to oval melanocytes and dendritic melanocytes in the middle and deep dermis, with foci of melanophages present in the deep dermis (Figure 1B, 1C). HMB45 and Mart-1 staining were positive in the melanocytic cells (data not shown). The patient underwent re-excision of the site with a 2- to 3-cm margin. Follow-up histopathology of the re-excised specimen, showed no evidence of residual melanoma. Routine laboratory studies and computed tomography scans of the chest, abdomen, and pelvis were negative. The patient is free of recurrence 1 year after the excision.

## Discussion

The clinical and pathological significance of melanocytic naevi lies principally in their relationship with melanoma. In the case of combined naevi the problem of a correct diagnosis is complicated by the presence of two or more different types of melanocytic naevi in a single lesion [4]. Overall, at dermoscopic examination naevus-associated melanomas display a lower number of features than *de novo *melanomas and are also less specific for malignancy [3]. This could be due to the incomplete vision of the neoplastic structures, partially obscured by the associated benign naevi. In the case presented, at dermoscopic examination only an atypical pigment network, feature present also in atypical benign naevi, was detectable with no areas of regression. The histopathological analysis, indeed, revealed that the melanoma was in a early phase of growth and this could account for the absence of regression areas at the dermoscopic examination. In conclusion, dermatologists should be aware that dermoscopic examination of combined naevi could not be exhaustive enough in order to exclude a diagnosis of malignancy. Therefore, these lesions should be always excised and histologically examined to achieve a conclusive diagnosis.

## Competing interests

The authors declare that they have no competing interests.

## Authors' contributions

AB and ED performed the surgical procedures and contributed to the analysis of the clinical data. PM performed the histological examination of the lesion. RM and PP contribuityed to the interpretation of the data and to the discussion of the manuscript AB performed the histological examination of the lesion and was a major contributor in writing the manuscript together with PM. All authors read and approved the final manuscript.

## Consent

Written informed consent was obtained from the patient for publication of this case report and accompanying images. A copy of the written consent is available for review by the Editor-in-Chief of this journal.

## References

[B1] Kaddu S, Smolle J, Zenahlik P, Hofmann-Wellenhof R, Kerl H (2002). Melanoma with benign melanocytic naevus component: reappraisal of clinicopathological features and prognosis. Melanoma Res.

[B2] Scolyer RA, Zhuang L, Palmer AA, Thompson JF, McCarthy SW (2004). Combined naevus: a benign lesion frequently misdiagnosed both clinically and pathologically as melanoma. Pathology.

[B3] Stante M, Carli P, Massi D, de Giorgi V (2003). Dermoscopic features of naevus-associated melanoma. Clin Exp Dermatol.

[B4] Granter SR, McKee PH, Calonje E, Mihm MC, Busam K (2001). Melanoma associated with blue naevus and melanoma mimicking cellular blue naevus: a clinicopathologic study of 10 cases on the spectrum of so-called "malignant blue naevus". Am J Surg Pathol.

